# Supplementary material for: Radar Multiple Bin Selection for Breathing and Heart Rate Monitoring in Acute Stroke Patients in a Clinical Setting
Source: Sensors (Basel). 2025 Dec 31;26(1):251. doi: 10.3390/s26010251 (PMC12788250; doi:10.3390/s26010251)
Supplement: Supplementary file 1 [file sensors-26-00251-s001.zip › sensors-4042671-supplementary.pdf]

Supplementary Material

for manuscript:

Radar Multiple Bin Selection for Breathing and Heart Rate Monitoring in Acute Stroke Patients in a Clinical Setting

Benedek Szmola, Lars Hornig, Jan Paul Vox, Thomas Liman, Andreas Radeloff, Birger Kollmeier, Karen Insa Wolf and Karsten Witt

Description

The Tables S1-S4 show the distribution of four breathing rate statistics on a patient level, to highlight the variation between individuals. Tables S5-S8 show the same statistics but for heart rate.

Table S1 – Breathing Rate: Recall (%). Distribution of per-patient values.

Range Bin Selection	Percentile					Min	Max
	10th	25th	50th	75th	90th		
Single	87.96	90.73	94.49	96.86	98.31	73.77	98.93
Multiple	40.36	65.03	81.18	87.39	93.71	3.72	98.00

Table S2 – Breathing Rate: Mean Absolute Error (BPM) . Distribution of per-patient values.

Range Bin Selection	Percentile					Min	Max
	10th	25th	50th	75th	90th		
Single	0.33	0.41	0.59	0.99	1.54	0.20	5.30
Multiple	0.17	0.24	0.36	0.50	0.77	0.14	2.94

Table S3 – Breathing Rate: Mean Absolute Percentage Error (%). Distribution of per-patient values.

Range Bin Selection	Percentile					Min	Max
	10th	25th	50th	75th	90th		
Single	2.04	2.59	4.16	6.62	10.34	1.22	39.86
Multiple	1.26	1.45	2.15	3.96	5.07	0.92	11.91

Table S4 – Breathing Rate: Spearman's Correlation Coefficient. Distribution of per-patient values.

Range Bin Selection	Percentile					Min	Max
	10th	25th	50th	75th	90th		
Single	0.64	0.70	0.80	0.86	0.89	0.24	0.95
Multiple	0.73	0.82	0.87	0.93	0.95	0.17	0.97

Table S5 – Heart Rate: Recall (%). Distribution of per-patient values.

Range Bin Selection	Percentile					Min	Max
	10th	25th	50th	75th	90th		
Single	63.97	74.12	85.79	93.27	95.11	47.47	96.82
Multiple	2.96	5.90	15.26	25.42	41.51	0.00	73.48

Table S6 – Heart Rate: Mean Absolute Error (BPM). Distribution of per-patient values.

Range Bin Selection	Percentile					Min	Max
	10th	25th	50th	75th	90th		
Single	0.94	1.38	2.10	5.19	12.49	0.62	23.36
Multiple	0.48	0.59	0.77	1.34	3.00	0.39	25.87

Table S7 – Heart Rate: Mean Absolute Percentage Error (%). Distribution of per-patient values.

Range Bin Selection	Percentile					Min	Max
	10th	25th	50th	75th	90th		
Single	1.42	1.97	3.49	8.73	25.07	1.06	51.81
Multiple	0.80	0.96	1.31	2.28	5.97	0.64	57.35

Table S8 – Heart Rate: Spearman's Correlation Coefficient. Distribution of per-patient values.

Range Bin Selection	Percentile					Min	Max
	10th	25th	50th	75th	90th		
Single	-0.09	0.26	0.65	0.85	0.93	-0.44	0.97
Multiple	0.50	0.83	0.90	0.95	0.97	-0.33	0.99
